# Genome-wide prediction of transcriptional regulatory elements of human promoters using gene expression and promoter analysis data

**DOI:** 10.1186/1471-2105-7-330

**Published:** 2006-07-04

**Authors:** Seon-Young Kim, YongSung Kim

**Affiliations:** 1 Human Genomics Laboratory, Genome Research Center, Korea Research Institute of Bioscience and Biotechnology, 52 Eoeun-dong, Yuseong-gu, Daejeon 305-333, Korea

## Abstract

**Background:**

A complete understanding of the regulatory mechanisms of gene expression is the next important issue of genomics. Many bioinformaticians have developed methods and algorithms for predicting transcriptional regulatory mechanisms from sequence, gene expression, and binding data. However, most of these studies involved the use of yeast which has much simpler regulatory networks than human and has many genome wide binding data and gene expression data under diverse conditions. Studies of genome wide transcriptional networks of human genomes currently lag behind those of yeast.

**Results:**

We report herein a new method that combines gene expression data analysis with promoter analysis to infer transcriptional regulatory elements of human genes. The Z scores from the application of gene set analysis with gene sets of transcription factor binding sites (TFBSs) were successfully used to represent the activity of TFBSs in a given microarray data set. A significant correlation between the Z scores of gene sets of TFBSs and individual genes across multiple conditions permitted successful identification of many known human transcriptional regulatory elements of genes as well as the prediction of numerous putative TFBSs of many genes which will constitute a good starting point for further experiments. Using Z scores of gene sets of TFBSs produced better predictions than the use of mRNA levels of a transcription factor itself, suggesting that the Z scores of gene sets of TFBSs better represent diverse mechanisms for changing the activity of transcription factors in the cell. In addition, cis-regulatory modules, combinations of co-acting TFBSs, were readily identified by our analysis.

**Conclusion:**

By a strategic combination of gene set level analysis of gene expression data sets and promoter analysis, we were able to identify and predict many transcriptional regulatory elements of human genes. We conclude that this approach will aid in decoding some of the important transcriptional regulatory elements of human genes.

## Background

With the genome sequences of many organisms completed, revealing the regulatory mechanisms of gene expression is the important aspect of genomics [1]. Recent innovative technologies such as microarray and chromatin immunoprecipitation combined with chip (ChIP – CHIP), and the whole genome sequencing of many organisms are producing enormous amounts of data that are useful in elucidating the transcriptional regulatory mechanisms of genes. Whole genome sequences provide information on the *cis*-acting regulatory elements of each gene. Gene expression data provide information on how the expression of each gene changes in a given condition, and the combination of chromatin immunoprecipitation (ChIP) with chip technology provides genome wide binding information concerning a transcription factor [2].

Many bioinformaticians have developed methods and algorithms for predicting transcriptional regulatory mechanisms from sequence data and gene expression data [3-6,8-12]. In one branch, a comparative sequence analysis of noncoding regulatory elements has helped to find new regulatory elements within many genes. New motifs have been discovered from evolutionarily conserved regions [13], from a list of co-regulated genes [14], or a list of functionally related genes [15,16]. Others have developed diverse algorithms that combine diverse sources of data to predict transcriptional regulatory mechanisms. To mention a few, Bussemaker et al. used a linear model to combine gene expression data with putative regulatory motifs and predicted significant regulatory elements [6]. Beer et al. [12] used probabilistic modeling in conjunction with diverse gene expression data and showed that regulatory elements can successfully predict the expression of certain genes. Bar-Joseph et al., and Gao et al. combined binding data with gene expression data to identify regulatory networks [7,9]. Others have inferred transcriptional elements by correlating the amount of transcription factor itself and its target genes [5,8,10].

However, most of above mentioned studied involved yeast which has much simpler regulatory networks than the human and has many genome wide binding data and gene expression data under diverse conditions [2,8,9,12]. Studies of genome wide transcriptional networks of human genomes are far behind those of yeast. A few studies reported on the development of tools that aids researchers in identifying putative transcriptional regulatory elements from a given gene expression study, but are not suitable for a meta-analysis of many gene expression studies [11,17].

Here, we report on a new computational method in which gene expression data analysis is combined with promoter analysis to infer the transcriptional regulatory elements of human genes. Our method is similar to Gao et al.'s approach in the use of correlation across multiple conditions [9], but is different in that this method used the composite expression of genes having the same predicted TFBSs rather than binding data which are available for only a few transcription factors in the human. The method, although simple in concept and calculation, was used to successfully identify many known TFBSs of genes and to predict many putative TFBSs that are worthy of further study.

## Results

### Algorithm

**Figure 1:**
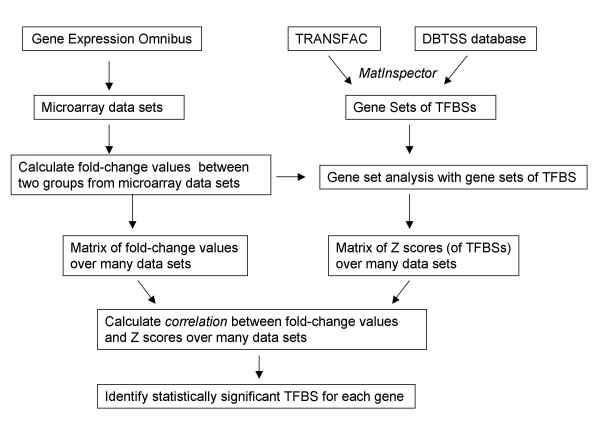
Schematic diagram of the procedures used in this study. Gene expression data sets were retrieved from GEO. Human promoters with experimentally verified transcription start sites were retrieved from the DBTSS database and analyzed for TFBSs with the TransFac version 3.0 database using the MatInspector program. For each microarray data set, the fold-change values between two experimental groups were calculated and used in gene set analyses with gene sets of TFBSs. This procedure was repeated over multiple data sets, resulting in a matrix of fold-change values over multiple data sets and a matrix of Z-scores over multiple data sets. The correlations between fold-change values and Z-scores over multiple data sets were calculated. Finally, statistically significant TFBSs were identified for each gene.

A flowchart of our algorithm is shown in Figure 1. Two important aspects are the calculation of the composite expression of genes having the same TFBS (referred to herein as Z score) using gene sets of TFBSs (a collection of genes having the same TFBS) as the first order analysis and a second order analysis in which the correlation between Z scores of gene sets of each TFBS and the fold-change values in gene expression for each gene over multiple microarray data sets is determined. The Z score is a normalized gene set enrichment score that describes the overall behavior of a gene set in a microarray data set [18].

### Analysis in one dataset

Given a microarray data set, there are two ways to infer which TFBSs are important for the observed changes in gene expression. One approach is to select genes that are significantly changed and then identify over-represented TFBSs among the promoters of those selected genes [14,17]. The other approach is to use changes in whole-genome expression and pre-defined gene sets of TFBSs to identify candidate TFBSs [19]. We based our method on this latter approach. To test whether our method could correctly identify important TFBSs from microarray data sets, we analyzed gene expression data for the response of human macrophages to treatment with HIV-1 gp120 [20]. The infection of macrophages by HIV-1 induces the expression of a variety of cytokines, chemokines, adhesion molecules, and apoptosis-related genes [20,21]. Among the many transcription factors involved in this response, NFκB is the most important [22]. Our analysis of the statistical significance of the 190 TFBSs used in the analysis showed that the NFκB binding sites (V$NFKB_C) had statistically significant Z scores (Table 1), thus showing that our approach could correctly detect important TFBSs in a given data set, as has been done in other similar approaches [19].

**Table 1:** Z-scores of TFBSs in an HIV-1 gp120 treated macrophage data set.

TFBS	Z score	p-value	q-value
V$NFKB_C	3.508	0	0.012
V$GATA1_04	2.617	0.009	0.082
V$NFKB_Q6	2.379	0.017	0.114
V$NFKAPPAB_01	2.177	0.029	0.169
V$NF1_01	2.148	0.032	0.176
V$NFKAPPAB65_01	2.131	0.033	0.177
V$TAXCREB_02	1.713	0.087	0.315
V$AP4_Q6	1.696	0.09	0.32
V$GATA1_01	1.524	0.128	0.409
V$OCT1_02	1.428	0.153	0.467

As another example of identifying TFBSs associated with specific biological conditions, we analyzed gene expression data sets for normal tissue expression. We analyzed the following data sets GDS181, GDS422-6, GDS596, GDS1985-8, and GDS1096 downloaded from the GEO site, and, as an example, show the result for GDS422-6 (Table 2) that contains expression profiles for 12 different normal tissues obtained using the Affymetrix U95A platform. To calculate tissue specific gene expression, we first calculated the mean expression of all 12 tissues; then, for each 12 tissues, we calculated the tissue specific gene expression by subtracting the mean expression of 12 tissues from the gene expression for each specific tissue. We proceeded as described above and identified a number of TFBSs that are associated with specific tissues. To name a few, TFBSs for hepatocyte nuclear factor-1 (HNF-1), HNF-4, and COUP-TF were identified in the liver specific gene expression profile, a TFBS for neuron restrictive silencer factor (NRSF) was identified in the brain and spinal cord gene expression profiles, and a TFBS for myocyte enhancer factor 2 (MEF-2) was identified in the skeletal muscle gene expression profile. Similar results were obtained when we analyzed the GDS181, GDS596, GDS1985-8, and GDS1096 data sets.

**Table 2:** Identification of TFBSs involved in tissue specific gene expression

TFBS_ID	TFBS	Tissue	Z score	p_value	q_value
V$NRSF_01	NRSF	brain	6.12	9.54E-10	1.62E-07
V$SRF_Q6	SRF	heart	4.63	3.68E-06	0.0007
V$SRF_C	SRF	heart	4.09	4.33E-05	0.00411
V$SRF_01	SRF	heart	3.74	0.0002	0.01151
V$MEF2_01	MEF-2	heart	3.47	0.0005	0.02441
V$HNF1_C	HNF-1	kidney	3.93	8.54E-05	0.01623
V$HNF1_01	HNF-1	liver	7.89	2.89E-15	3.28E-13
V$GRE_C	GR	liver	4.85	1.22E-06	6.91E-05
V$HNF4_01	HNF-4	liver	4.39	1.15E-05	0.00043
V$HNF1_C	HNF-1	liver	3.72	2.00E-04	0.00559
V$COUP_01	COUP-TF, HNF-4	liver	3.5	5.00E-04	0.01059
V$AP1_C	AP-1	lung	4.13	3.64E-05	0.00682
V$NFKAPPAB65_01	NF-κB	lung	3.66	0.0002	0.0233
V$AP1_Q6	AP-1	prostate	3.67	0.0002	0.02802
V$MEF2_02	MEF-2	skeletal muscle	4.53	5.96E-06	0.00099
V$NRSF_01	NRSF	spinal cord	3.87	0.0001	0.02026
V$IRF1_01	IRF-1	spleen	3.99	6.70E-05	0.00936
V$NFKAPPAB65_01	NF-KB	spleen	3.81	0.0001	0.00985
V$NFKAPPAB_01	NF-κB	spleen	3.55	0.0004	0.01815
V$E2F_02	E2F	thymus	4.65	3.29E-06	0.00043
V$CETS1P54_02	c-Ets-1	thymus	4.18	2.93E-05	0.00126
V$IRF1_01	IRF1	thymus	4.2	2.65E-05	0.00126
V$E2F_01	E2F	thymus	4.09	4.39E-05	0.00142
V$CETS1P54_01	c-Ets-1	thymus	4.01	5.98E-05	0.00155
V$E2F_Q6	E2F	thymus	3.74	0.0002	0.00396
V$NRF2_01	NRF-2	thymus	3.58	0.0003	0.00564
V$OCT1_07	Oct-1	thymus	3.17	0.0015	0.01993
V$OCT1_05	Oct-1	thymus	3.17	0.0015	0.01993
V$NFY_Q6	NF-Y	thymus	2.85	0.0043	0.04663

### Analysis in multiple data sets

**Figure 2:**
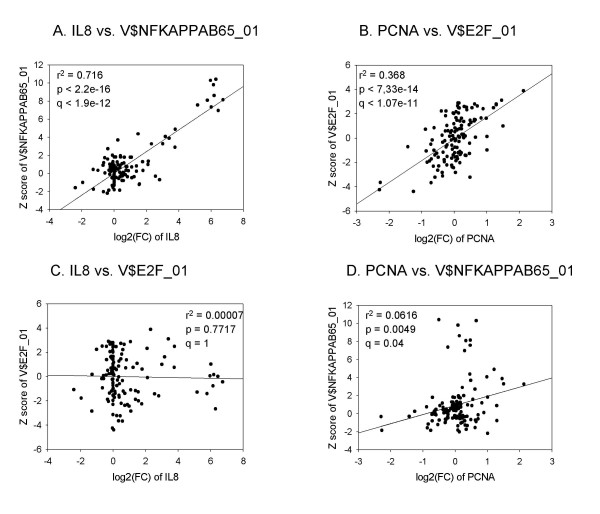
Patterns of correlation between the fold-change values and Z-scores of TFBSs over multiple data sets for *IL8 *and *PCNA*. **a**. Correlation between the fold-change values for IL8 and Z-scores of V$NFKAPPAB65_01 over 127 microarray data sets. **b**. Correlation between the fold-change values for *PCNA *and Z-scores of V$E2F_01. **c**. Correlation between the fold-change values for IL8 and Z-scores of V$E2F_01. **d**. Correlation between the fold-change values for IL8 and Z-scores of V$E2F_01. The Pearson's correlation coefficient was used to calculate the degree of correlation between the two arrays and the *t*-test was used to evaluate the significance of the correlation (see Methods).

We next tested whether the second-order analysis designed to observe a correlation between the fold-change values in gene expression and Z scores of the TFBSs over multiple data sets could be used to correctly identify important regulatory elements of a gene. Two well-known genes, *IL8 *and *PCNA*, are shown as examples (Figure 2). *IL8 *encodes a chemokine that is induced in diverse cell types in response to inflammation, mainly through the activation and nuclear translocation of NFκB [23]. *PCNA *is a cofactor of DNA polymerase delta that plays a role in increasing the processivity of leading strand synthesis during DNA replication and is mainly regulated by E2F1 transcription factors [24]. The analysis of the fold-change values of *IL8 *and the Z scores of TFBS V$NFKAPPAB65_01 over 127 independent data sets yielded a significant correlation (r^2 ^= 0.716, p < 2.2 × 10^-16^, q < 1.9 × 10^-12^; Pearson correlation test) (Figure 2A). The fold- change values of *PCNA *and the Z scores of V$E2F_01, the TFBS for E2F1 transcription factors, were also significantly correlated (r^2 ^= 0.368, p < 7.33 × 10^-14^, q < 1.07 × 10^-11^) (Figure 2B). On the contrary, the correlation between the fold change values of IL8 and the Z scores of the TFBS V$E2F_01 was insignificant (Figure 2C). The correlation between the fold changes of PCNA and the Z scores of V$NFKAPPAB65_01 showed a modest significance (r^2 ^= 0.0616; p < 0.0049; q < 0.04) (Figure 2D). These data show that it is possible to identify the main TFBS of a gene by correlating the fold-change values of the gene and the Z scores of TFBSs over multiple independent microarray data sets.

### Selection of the optimal overall similarity cut-off value for each MatInspector position weight matrix (PWM)

**Figure 3:**
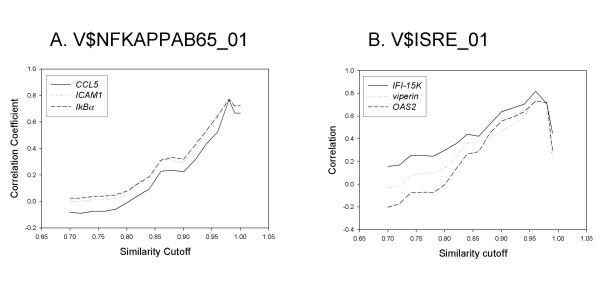
Selection of optimal matrix similarity cut-off value for each TFBS. Two TFBSs (V$NFKAPPAB65_01 and V$ISRE_01) are shown as examples. When predicting putative TFBSs from promoter sequences using the MatInspector program, the core similarity cut-off value was set as 0.75, and the overall similarity cut-off value was varied from 0.7 to 1.0 by increments of 0.02.

The reliability of predicting TFBSs in a promoter sequence depends on the cut-off values of the overall similarity of the position weight matrix (PWM). Less stringent cut-off values yield more false-positive predictions but fewer false-negative predictions. Most previous studies have applied two or three different cut-off values (i.e., 0.8, 0.85, and 0.9) to all TFBSs unequivocally. We addressed this issue by applying a wide range of cut-off values for the overall matrix similarity for each TFBS and then observed the degree of correlation in multiple microarray data sets. As expected, we found that the choice of cut-off values had a significant effect on the overall performance of our algorithm. Two examples, using the TFBSs V$NFKAPPABP65_01 and V$ISRE_01, demonstrate this point (Figure 3). When we varied the overall matrix similarity from 0.7 to 1.0 in increments of 0.02, the correlation coefficient was the highest when the cut-off value for the overall matrix similarity was 0.98 for V$NFKAPPAB65_01 and 0.96 for V$ISRE_01. The optimal cut-off value for each TFBS varied widely from 0.72 (i.e. V$P53_01) to 1.00 (i.e., V$CREB_01). The cut-off values for all TFBSs used in this study and the number of promoters predicted for each cut-off value is listed in Additional file 2.

### Estimation of the accuracy of TFBSs prediction by comparing with known transcription regulatory elements in TRED database

Our method predicted many TFBSs for each gene (8845 genes in the U95A data set and 12803 genes in the U133A data set). The overall prediction rate was 21.4% (U95A) and 21.5% among a total of 190 * 8845 TFBSs (U95A) and 190 * 12803 genes (U133A) at a false discovery rate (q-value) of 0.05. We estimated the accuracy of predicting the TFBSs by a comparison of known transcriptional regulatory elements in the TRED database that contains gene transcriptional regulation information including TFBSs with available experimental evidence [25]. Among the four levels of experimental evidence (known, likely, maybe, and predicted) in the TRED database, we used only 'known' evidence that was validated by a literature search [25].

Among 5004 known TFBSs in 1724 genes in the TRED database, there were 2721 known TFBSs in 1366 genes in the U95A platform and 2847 known TFBSs in 1450 genes in the U133A platform. We applied a *t*-test to each correlation coefficient calculated in our analyses to infer the significance of each TFBS prediction and applied the false discovery rate method to adjust the *p*-values produced in multiple hypothesis testing. A *q*-value below 0.05 was regarded as significant and the percentage of successful predictions was calculated. 43.1% of 2721 known TFBSs in 1366 genes were predicted for the U95 data set and 43.9% of 2847 known TFBSs in 1450 genes were predicted for the U133 data set (Table 3).

**Table 3:** Accuracy of the prediction of TFBSs: comparison with known TFBSs of genes.

		Matrix				Factor		
Platform	U95		U133		U95		U133	
q_value	count	percent	count	percent	count	percent	count	percent

0.001	378	13.4	390	13.2	185	6.6	218	7.4
0.005	610	21.6	659	22.3	311	11.1	347	11.8
0.01	773	27.4	800	27.1	389	13.9	436	14.9
0.05	1216	43.1	1295	43.9	722	25.8	787	26.8
0.1	1524	54.1	1650	56.0	1003	35.9	999	34
1	2819	100	2949	100	2797	100	2935	100

Previous studies have suggested that gene Y contains a binding site for transcription factor X if gene expression changes of transcription factor X and gene expression changes of gene Y are significantly correlated with each other over multiple data sets [5,10,26]. Our method is different from previous studies in that the Z scores of gene sets of TFBSs rather than changes in gene expression of each TF were used. To determine how our method performs compared with previous methods, we evaluated the performance of the method for predicting transcriptional regulatory elements from a correlation between the gene expression changes of each TF and each gene using the same data sets and a statistical testing procedure. We found that 25.8% of the known TFBSs among 1366 genes were predicted for the U95 data set and 26.8% of known TFBSs among 1450 genes were predicted for the U133 data set (Table 3).

### Evaluation by comparing two independent predictions from two different data sets

**Figure 4:**
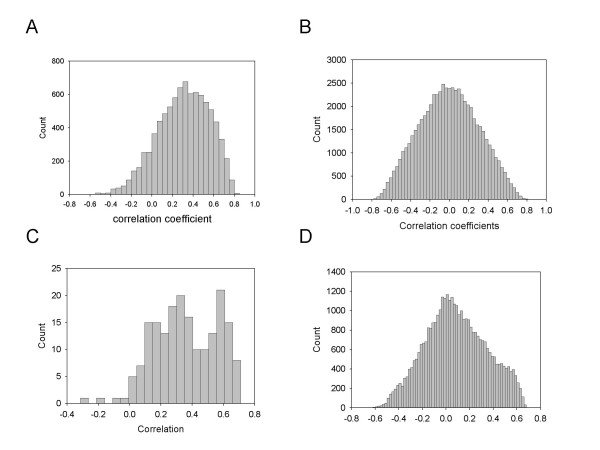
Distribution of correlation coefficients between two independently prepared predictions of TFBSs. A. Distribution of correlation coefficients of 8738 genes between 190 TFBSs predictions from U95A and 190 TFBSs predictions from U133A data sets. B. Distribution of correlation coefficients of 8738 genes between two groups each of which was randomly selected from a standard normal distribution. C. Distribution of correlation coefficients of 190 TFBSs between 8738 predictions from U95A and 8738 predictions from U133A data sets. D. Distribution of correlation coefficients of 190 TFBSs between two groups each randomly selected from standard normal distribution.

In the second evaluation, we analyzed the degree of correlation between the predicted TFBSs from U95A data sets and the predicted TFBSs from U133A data sets. We assumed that a good correlation between predictions from independent microarray data sets would further support the reliability of our algorithm. To ensure independency between the U95A and U133A microarray data sets, we removed data sets for common biological events and used the remaining data sets. We specifically removed GDS156, GDS817, GDS854, and GDS915 from the U95A data sets and GDS287, GDS472, GDS820, GDS855, and GDS914 from the U133A data sets (Additional file 1). We compared the predictions made from U95A and U133A data sets in two dimensions; one being a gene-to-gene comparison and the second a TFBS-to-TFBS comparison. As expected, we found a high percentage of significant correlations between the predicted TFBSs from U95A and the predicted TFBSs from U133A data sets (Figure 4A and 4C). On the contrary, two independent data sets that were randomly sampled from a normal distribution showed a pattern of distribution for correlation coefficients that were similar to the standard normal distribution (Figure 4B and 4D). Specifically, 74.3% of 8728 genes showed a significant correlation at a p-value of 0.05, while only 4.9% of 8728 genes showed significant correlation at a p-value of 0.05 (Table 4).

**Table 4:** Evaluation by comparing two independent predictions using two different data sets

U95A vs. U133A				Random Control
p value	cumulative count	cumulative percent	cumulative count	cumulative percent

0.0001	4929	56.5	0	0
0.001	5450	62.4	10	0.11
0.005	5836	66.9	42	0.48
0.01	6022	69	85	0.97
0.05	6486	**74.3**	430	**4.9**
0.1	6699	76.8	805	9.2
0.2	6937	79.5	1722	19.7
0.3	7095	81.3	2614	29.9
0.4	7226	82.8	3450	39.5
0.5	7319	83.9	4365	50
0.6	7400	84.8	5241	60
0.7	7478	85.7	6112	69.9
0.8	7542	86.4	6988	80
0.9	7616	87.3	7896	90.4
1	8728	100	8728	100

### Examples of a genome-wide prediction of TFBSs: NFκB

**Figure 5:**
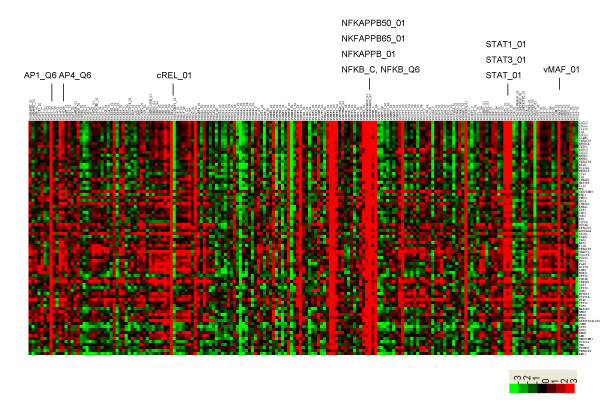
Identification of NFκB-regulated genes by selecting significant correlations between the fold-change values of the genes and Z-scores of V$NFKAPPAB65_01 among multiple data sets. Correlation coefficients were converted into *t*-scores. Java Treeview was used to represent visually the matrix of *t*-scores over all TFBSs and genes. Genes that correlated highly with V$NFKAPPAB65_01 are shown. Several TFBSs showing high correlation with genes regulated by V$NFKAPPAB65_01 are marked.

To demonstrate biological significance of our predictions, a TFBS for NFκB is shown as an example from the 190 TFBSs examined. The list of genes that are significantly correlated with V$NFKAPPAB65_01 over 127 independent data sets is shown in Figure 5 and Additional file 3. The genes in the list include those encoding various chemokines (*CCL20, CXCL1, CXCL2, CXCL3*, *CCL2*, *CXCL5*, and *IL8*), cytokines (*IL6*), adhesion molecules (*ICAM*), NFκBs (*NFKBIA, NFKB1*, and *NFKB2*), and interferon-induced genes (*INFAR2*, *IRF1*, *TNFAIP3, and TNFAIP2*, etc.), all of which are known targets of *NFκB*, along with many genes that are not currently known as *NFκB *regulated genes (data now shown) [16]. In addition, we found that many genes showing a high correlation with V$NFKAPPAB65_01 also showed a significant correlation with TFBSs, such as V$AP1_Q6, V$AP4_Q6, V$STAT1_01 and V$STAT3_01 (Figure 5). V$AP1 and V$STAT1 are TFBSs that are over-represented among the *NFκB*-regulated genes, and thus were included in models for *NFκB*-regulated, immunologically related gene promoters [16]. These results show that our approach was able to identify, in addition to individual TFBSs, *cis*-regulatory modules (CRMs) of each gene, (i.e., collections of TFBSs that function together to regulate the expression of a gene).

## Discussion

In this study, a computational approach is proposed for predicting the transcriptional regulatory elements of individual human genes using both gene expression data sets and promoter sequences in a genome-wide manner. Our approach uses our recently developed tool, parametric analysis of gene set enrichment, which produces a Z score which is useful in the analysis of multiple gene expression data sets.

An important issue encountered in predicting TFBSs from a promoter sequence with position weight matrices is to select an optimal cut-off value for a matrix similarity. Previous researchers, although recognizing its importance, didn't systematically select optimal cut-off values for each TFBS, but applied merely two or three different levels of cut-off values (for example, 0.8, 0.85, and 0.9) to all TFBSs [17]. When we varied the cut-off values from 0.7 to 1.0 in increments of 0.02, we found that the optimal cut-off value for each TFBS varied widely from 0.72 to 1.00, showing the importance of a systemic approach in the selection of optimal cut-off values (Figure 3 and Additional file 2). However, a few points are worth mentioning. First, there may be a concern that using the most stringent cut-off value would lead to a smaller number of genes in a gene set and, as a result, predicted TFBSs would be restricted only to those which were used to create the initial gene sets (a circularity problem). We tested whether such a problem actually occurs but found that many TFBSs that were not included in the initial gene set were predicted even when the most stringent cut-off value was used (data not shown). Second, in cooperative binding, which is prevalent in many cooperating transcriptional modules, one factor can have an especially weak binding site escaping any type of statistical detection. When the most stringent cut-off value is used, our method is likely to miss this weak binding site. Thus, it may be helpful to try a few less stringent cut-off values to avoid missing weak binding sites or reduce false negative predictions.

Another important point when transcriptional regulatory networks are inferred from gene expression data is that many transcription factors (TFs) are regulated by posttranscriptional as well as transcriptional mechanisms. Thus, some TFs exert their altered activity on target genes through changes in the amount of their mRNA, while other TFs utilize other mechanisms such as nuclear translocation, phosphorylation, proteolytic degradation, or interaction with small ligands [12]. Therefore, recent studies that have focused on TFBSs itself rather than TFs have enjoyed great success [12]. To determine which methods are better in identifying human transcriptional regulatory networks, we compared two kinds of measures of transcriptional activity, Z scores of gene sets of TFBSs and the amount of TF mRNA itself. Our results showed better performance for the Z scores of gene sets of TFBS over TF mRNA levels (Table 3), suggesting that Z scores of gene sets of TFBS might reflect diverse mechanisms in changes in TFs in the cell and might be better suited to infer transcriptional networks than the amount of TF mRNA.

We computationally validated our prediction of TFBSs by observing the number of experimentally known TFBSs in the TRED database that could be predicted by our method. While we used TRED database in this work, we should mention that there are more complete, literature-based, but commercial, databases (for example, Genomatix Suite) available. The results of validation of predicted TFBSs using the TRED database showed a successful prediction rate of 43.1% (U95A) and 43.9% (U133A) at a false discovery rate (q-value) of 0.05. This corresponds to false negative rate of 56.9% and 56.1%. The second validation (Figure 4, Table 4) analysis showed that our method for predicting TFBSs from gene expression data was able to extract real signals from noise irrespective of the data set used. The two data sets (U95A and U133A) we used were from different platforms, have different gene contents, and, above all, involved different experimental conditions, but showed high correlations with each other between TFBSs predictions. This suggests that it is possible to consistently infer transcriptional regulatory elements, irrespective of the data sets used. This also suggests that cells use a limited number of transcription regulatory elements to adjust themselves to diverse environmental conditions. The combinatorial nature of transcription factors is one way to ensure an effective adaptation to diverse conditions, and is utilized in many genes. Many researchers have applied the combinatorial nature of transcription factors to the computational prediction of transcriptional networks with great success [12,27]. We plan to adopt the combinatorial analysis to our method and expect to further improve this method.

Many genes are regulated by different TFBSs under different conditions. With enough data sets in diverse conditions, our approach should identify different TFBSs under different conditions in regulating gene expression. We tested if our approach was able to identify different TFBSs under different conditions on a few selected genes and actually found that phenomena (data not shown). At present, we didn't systematically analyze the two data sets (U95A and U133A) to identify such condition-dependent TFBSs because data sets included in this study didn't have diverse experimental conditions, but we think that identifying condition-dependent TFBSs is an important work that should be achieved when enough data sets are available.

We understand that a successful prediction rate of 43.1% and 43.9% is far from satisfactory, but considering several limitations in our approach, the method is promising. To mention a few limitations, we restricted our analysis of promoter sequence to 1200 bp (between -1000 bp and +200 bp relative to the transcription start site) of a gene, but many regulatory elements in human genes, in contrast to yeast genes, reside outside this proximal promoter region. The second limitation is that a sufficient number of gene expression data sets are not currently available to include the diversity of conditions needed. We used 127 and 138 conditions with two platforms, while yeast researchers are able to use more than 1,000 conditions in a computational study [28].

## Conclusion

A correlation between the Z scores of gene sets of TFBSs, produced by gene set analysis, and the fold changes in gene expression across multiple conditions permitted successful identification of many functionally important TFBSs of human genes. We successfully identified many known TFBSs of human genes and predicted numerous TFBSs of genes that are worthy of further study. We also showed that the Z scores of gene sets of TFBSs better represented changes in the activity of TFs in a cell than transcription factor mRNA itself. In a single gene expression data set, our method was able to identify transcription regulatory elements that caused the gene expression changes that are observed for many genes. Elucidating the regulatory elements of entire genomes is the next important task in genomics and requires innovations in both experimental techniques and computational methods. We hope our approach will aid in decoding the important transcriptional regulatory elements of genes by strategically combining gene expression data with genomic sequence data.

## Methods

### Promoter and prediction of TFBS

Human promoter sequences for which transcription start sites are accurately known were downloaded from the DBTSS (Database of Transcriptional Start Sites) [29] which contains upstream sequences at -1000 to +200 relative to the transcription start site [30]. TFBSs were predicted using the MatInspector program with position weight matrices (PWMs) of the TransFac database (ver. 3.0) included in the MatInspector program [31]. We set the MatInspector core similarity to 0.75 and varied the overall matrix similarity from 0.7 to 1.0 in 0.02 increments for each TFBS. We used TRED (Transcriptional Regulatory Element Database) database [32] to obtain a list of known transcriptional regulatory elements for genes [25].

### Gene expression data sets and data analysis

The microarray data sets used in this study were downloaded from the Gene Expression Omnibus (GEO) website [33]. We used only data sets calculated using MAS5 (microarray suite 5) algorithm to ensure the same processing of all data sets [34]. The list of data sets is given in Additional file 1. The microarray data set describing the gene expression changes of macrophages treated with HIV-1 gp120 was generously provided by Dr. Cicala [20]. We used data sets GDS181, GDS422-6, GDS596, GDS1985-8, and GDS1096 to study gene expression in normal human tissues. Each data set was analyzed as follows. First, each sample within a gene expression data set (GDS) was normalized by the global mean of each sample to obtain a global mean of 1000. Signal values lower than 100 were then increased to 100 and the log base 2 was taken. All subsequent calculations were done using log2-transformed values. For a gene with multiple probes, we took the mean value of the multiple probes. Our data sets encompassed various experimental conditions including a comparison between two groups (for example, tumor vs. normal), a comparison among multiple conditions, and time course experiments. We calculated the log2-transformed fold change values between two groups. When one data set had multiple experimental conditions, each condition was regarded as a separate data set in calculating the log2-transformed fold change values. We chose to analyze data sets of the Affymetrix U95A or U133A platforms because many data sets are available for those two platforms.

### Parametric analysis of gene set enrichment

One hundred ninety different gene sets for TFBSs were constructed from the predicted TFBSs for 14776 human promoters. We calculated the composite expression of genes having the same predicted TFBS (hereafter referred to as the Z score) for each TFBS in each microarray data set using gene set analysis [18]. The Z score in our analysis is defined as



, where *X *is the mean of fold change values of genes having the same predicted TFBS, *μ *the mean of fold change values of total genes in a data set, and *δ *the standard deviation for the fold change values of total genes in a data set, and *n *the size of the gene set. The Z score serves as a measure of how far the composite expression of genes having the same predicted TFBS deviates from the mean of the fold change values of the total genes in a given data set. The correlation between Z scores and fold change values among multiple microarray data sets was calculated using Pearson's correlation coefficient. The significance of each correlation coefficient was inferred from a *t*-test using the following mathematical formulae.

When the number of samples is *n *and Pearson's correlation coefficient is *r*:



The statistical significance of the *t*-value is evaluated using the *t*-test with *n*-2 degrees of freedom [9,35]. One possible concern in our approach is that the Z score and the fold change for a gene expression data for which is included in the calculation of the Z score are, strictly speaking, not independent variables. However, because each gene set is large (see Additional file 2), we consider that this lack of independence is not a serious practical concern. Java Treeview was used to visually represent the matrix of *t-*scores over all TFBSs and genes[36]. The method of false discovery rates was used to adjust p values for multiple hypothesis testing [37]. The adjusted q values were calculated using the qvalue package of the Bioconductor project [38].

### Validation of predicted TFBSs

We validated our predicted TFBSs in two ways. We first calculated the number of known transcriptional regulatory elements of genes in the TRED database that could be successfully predicted by our method [25]. Second, we used two independent gene expression data sets (U95A and U133A) in predicting the TFBSs, and compared the extent to which two predictions were correlated with each other.

## Authors' contributions

SYK designed the algorithm, collected the data sets, performed bioinformatics analyses, and drafted the manuscript. YSK designed the algorithm and wrote the manuscript. All the authors have read and approved the final manuscript.

## Supplementary Material

Additional File 1 List of data sets used in this study. Click here for file

Additional File 2 Number of predicted TFBSs at each overall matrix similarity cut-off value. Click here for file

Additional File 3 List of genes that are highly correlated with V$NFKAPPAB65_01. Click here for file
